# Intrinsic Functional Plasticity of the Thalamocortical System in Minimally Disabled Patients with Relapsing-Remitting Multiple Sclerosis

**DOI:** 10.3389/fnhum.2016.00002

**Published:** 2016-01-25

**Authors:** Fuqing Zhou, Honghan Gong, Qi Chen, Bo Wang, Yan Peng, Ying Zhuang, Chi-shing Zee

**Affiliations:** ^1^Department of Radiology, The First Affiliated Hospital, Nanchang University, Nanchang, China; ^2^Department of Burns, The First Affiliated Hospital, Nanchang University, Nanchang, China; ^3^Department of Oncology, The Second Hospital of Nanchang City, Nanchang, China; ^4^Department of Radiology, University of Southern California, Los Angeles, CA, USA

**Keywords:** functional connectivity, structural connectivity, thalamus, relapsing-remitting multiple sclerosis, fiber tractography

## Abstract

The thalamus plays a crucial role in sensorimotor, cognitive, and attentional circuit functions. Disruptions in thalamic connectivity are believed to underlie the symptoms of multiple sclerosis (MS). Therefore, assessing thalamocortical structural connectivity (SC) and functional connectivity (FC) may provide new insights into the mechanism of intrinsic functional plasticity in a large-scale neural network. We used resting-state FC measurement and diffusion tensor imaging probabilistic tractography to study the functional and structural integrity of the thalamocortical system in patients with relapsing-remitting MS (RRMS) and matched healthy controls. In the thalamocortical connections of RRMS patients, we found lesion load-related regional FC in the right temporal pole, which reflected compensatory hyperconnectivity related to lesion-related demyelination. We also found significant correlations between increased diffusivity and slowed cognitive processing (PASAT) or the impact of fatigue (MFIS-5), as well as between connective fiber loss and disease duration. Taken together, the evidence from SC and FC analysis of the thalamocortical system suggests that minimally disabled RRMS patients exhibit a dissociated SC–FC pattern and limited regional functional plasticity to compensate for the chronic demyelination-related loss of long-distance SC. These results also provide further evidence supporting the notion that MS is a disorder of anatomical disconnection.

## Introduction

Pathological (Geurts and Barkhof, 2008; Vercellino et al., 2009; Haider et al., 2014) and neuroimaging (Ramli et al., 2010; Zhou et al., 2010; Bodini et al., 2011) studies have demonstrated the widespread involvement of gray matter (GM) and white matter (WM) of the brain in multiple sclerosis (MS). In MS, initially minimal structural damage can be sufficient to trigger a strong response or to diminish function (Roosendaal et al., 2010; Faivre et al., 2012) because anatomical structures have been considered to the underlying basis for functions. The disruption of long-distance WM connectivity may greatly impact the integrity of network function (Zhou et al., 2014a).

The thalami are widely interconnected with the cerebral cortex via long-distance WM tracts (referred to as the connectivity “backbone”), and the thalami play a central role in the sensory relay, limbic, and other important functional systems. Growing evidence pertaining to MS has shown substantial thalamic degeneration (Rapalino, 2009; Tovar-Moll et al., 2009; Cappellani et al., 2014) even in pre-MS patients presenting with a clinically isolated syndrome (CIS) (Henry et al., 2009). Different local thalamic alterations, including thalamic microstructural damage (Tovar-Moll et al., 2009), neuronal loss (Cifelli et al., 2002; Haider et al., 2014), increased intrinsic oscillation amplitude (Zhou et al., 2014b), and increased intrathalamic and interthalamic connectivity (Liu et al., 2015b), as well as varying alterations of thalamic connectivity, including thalamocortical structural disconnection (Bisecco et al., 2015) and increased thalamocortical functional connectivity (FC) associated with decreased performance (Tona et al., 2014), have been described in MS. However, other studies have reported conflicting findings related to thalamocortical FC (Liu et al., 2015a,b). By comparison, relatively little is known about the relationship between FC and structural connectivity (SC) with respect to the thalamocortical connections in minimally disabled patients with relapsing-remitting MS (RRMS). In addition, the thalamocortical system in RRMS patients is an excellent paradigm for long-distance (interregional) connectivity. Understanding the thalamocortical system may be particularly important for diseases of the brain. In the current study, we hypothesized that both thalamocortical SC and FC, as well as their correlations with clinical indices, are disrupted in RRMS. To test this hypothesis, probabilistic tractography and resting-state FC measurements were combined to characterize the SC of long WM tracts and FC based on the coherence of intrinsic neuronal activity in paired thalamic connectivity-defined regions (CDRs) and exclusive cortical regions (ECRs). Furthermore, we explored the coupling mechanisms of thalamocortical connectivity that might underlie the observed alterations in SC and FC. Studying the relationship between thalamocortical SC and FC may provide new insights into the neural underpinnings of RRMS.

## Materials and Methods

### Participants

Seventy-eight patients with clinically definite MS at the First Affiliated Hospital of Nanchang University participated in this study from May 2010 to June 2015. All patients received a series of clinical and MRI examinations, including the expanded disability status scale (EDSS). The paced auditory serial addition test (PASAT) was used to obtain data related to cognitive processing speed, and the modified fatigue impact scale (MFIS-5) was examined to measure the impact of fatigue on physical, cognitive, and psychosocial functioning. After diagnosis, the recruited patients were confirmed to exhibit the following characteristics: RRMS course (Hurwitz, 2009); EDSS score <2.5, which corresponds to minimal disability (Kurtzke, 2008); lacking visible lesions in the bilateral thalamus, which corresponds to a normal-appearing thalamus; and treatment with immunomodulatory medication. None of the recruited patients experienced any relapses, which were identified by follow-up neurological assessments and contrast-enhanced MRI after relapse onset (12 weeks), or received corticosteroid treatment during the month preceding MRI acquisition. Healthy control (HC) participants from the local community were individually matched to the patients by gender, age, education level, and lack of history of neurological or psychiatric disorders. After excluding patients who had excessive head motion during scanning [see the *Functional Data Preprocessing* section] and those who did not meet the selection criteria, 20 RRMS patients (18 patients receiving β-interferons; 2 receiving Glatiramer acetate) and well-matched HC subjects were included in this study.

This study was performed according to approved guidelines and was conducted in compliance with the principles of the Declaration of Helsinki; additionally, this study was approved by the Medical Research Ethics Committee and the Institutional Review Board of the First Affiliated Hospital of Nanchang University. All subjects signed written consent forms for participation in this study.

### MRI Data Acquisition

All participants were scanned using a 3.0-T MRI scanner (Trio Tim, Siemens Medical Systems, Erlangen, Germany). In this study, T_2_-weighted imaging (*T*_2_*WI*), T_1_-weighted imaging (*T*_1_*WI*), diffusion tensor imaging (*DTI*), and rs-fMRI were acquired as follows: (1) turbo spin-echo sequences for the *T*_2_*WI* scan [repetition time (TR)/echo time (TE) = 5100/117 ms; field of view (FOV) = 240 mm × 240 mm; matrix = 416 × 416; slice number = 22; slice thickness = 6.5 mm; echo train length = 11; number of excitations (NEX) = 3; and orientation = axial]; (2) three-dimensional magnetization-prepared rapid acquisition gradient-echo (3D MPRAGE) sequences for the high-resolution *T*_1_*WI* scan (TR/TE = 1900/2.26 ms; FOV = 215 mm × 230 mm; matrix = 240 × 256; slice number = 176; slice thickness = 1.0 mm; NEX = 1; and orientation = sagittal); (3) spin-echo single-shot echo planar imaging (EPI) for the *DTI* scan [TR/TE = 7200/104 ms; FOV = 230 mm × 230 mm; matrix = 128 × 128; slice number = 49; slice thickness = 2.5 mm; NEX = 2; orientation = axial; and 64 non-linear diffusion weighting gradient directions with *b* = 1000 s/mm^2^ and 1 additional image without diffusion weighting (i.e., b = 0 s/mm^2^)]; (4) an EPI sequence for the rs-fMRI scan (TR/TE = 2000/30 ms; FOV = 200 mm × 200 mm; matrix = 64 × 64; flip angle = 90°; 30 interleaved axial slices; slice thickness = 4 mm; interslice interval = 1.2 mm; and number of time points = 240). During rs-fMRI scanning, all subjects were instructed to keep their eyes closed, not to think about anything in particular, and not to fall asleep; and (5) axial conventional *T_*1*_*WI**, *T_*2*_*WI**, *T_*2*_*-FLAIR** (fluid-attenuated inversion recovery), and contrast-enhanced *T_*1*_*WI** were acquired in the brain for the diagnosis in each subject. A foam pad was used to minimize the head motion of all subjects. At the end of the scanning sessions, all the participants reported that they had not fallen asleep during the scan.

### Thalamic, Lesion Load, and Brain Atrophy Measurements

The bilateral thalamic boundaries were determined manually from high-resolution *T*_1_*WI* scans by an experienced neuroradiologist (F.Z., with 6 years of experience) who was blinded to the clinical information using MRIcron^1^. For normalization, the thalamic fraction (TF) was calculated as the ratio of raw thalamic volume to the intracranial volume for each subject.

All visible lesions were identified from the *T_*2*_*WI**, and a binary lesion mask was manually drawn by an experienced neuroradiologist (F.Z.) using MRIcron. The *T_*2*_*WI** was coregistered with the T1-weighted structural image. Transformation of the aforementioned *T_*1*_*WI**-based individual brain according to the Montreal Neurological Institute (MNI) standard brain dimensions was used to warp the lesion mask into the MNI space. The lesion load calculated from the spatially normalized lesion mask [as the normalized total WM lesion load (TWMLL)] reflected the TWMLL relative to the standard MNI brain volume rather than the individual brain volume so that the effects of ­differences in brain volume were controlled (Pelletier et al., 2004; Shu et al., 2011).

To test the reproducibility of our findings, thalamic volume and lesion load were measured on two separate occasions (at least 3 months apart) in the patients, and the inter-rater reliabilities were 93.2 and 92.6%, respectively.

The high-resolution *T*_1_*WI* data were segmented into GM, WM, and CSF using the new segmentation algorithm provided in Statistical Parametric Mapping 8 (SPM8^2^). The GM and WM probability images were then registered and warped into MNI space using the aforementioned DARTEL process. The brain parenchymal fraction (BPF) was then calculated as the ratio of the brain parenchymal (GM and WM) volume to the intracranial volume.

### Characterization of Thalamocortical FC

#### Functional Data Preprocessing

The rs-fMRI data were preprocessed using the Data Processing Assistant for Resting-State fMRI Advanced Edition (DPARSFA) V3.1^3^ in SPM8 running in Matlab 2014b (MathWorks, Natick, MA, USA). rs-fMRI data preprocessing included the following steps. The first 10 time points were removed, and the remaining time points were processed according to slice timing, voxel-specific head motion calculation [according to the criteria of Van Dijk et al. (2012); between-group differences are shown in Table 1] and correction to adjust the time series. Additionally, subjects with head movement in the cardinal directions (*x*, *y*, *z*) of >2 mm and a maximum rotation (*x*, *y*, *z*) of >2° were excluded. Then, the corrected images were used for unified segmentation of the *T_*1*_*WI** for non-linear registration and normalization of the functional images to the MNI space with 3 × 3 × 3 mm^3^ re-sampling. Next, spatial smoothing was then performed using a 6-mm full-width–half-maximum Gaussian kernel. Temporal band-pass filtering (0.01 Hz < *f* < 0.08 Hz) was used to reduce low-frequency drift and physiological high-frequency noise. Finally, multiple regression was performed to remove the effects of nuisance covariates from the BOLD data, which included a ventricular signal averaged from ventricular regions of interest (ROIs), a WM signal averaged from WM ROIs, a global signal averaged across the entire brain, six head realignment parameters obtained by rigid body head motion correction, the derivatives of each of these signals, and a voxel-specific head motion parameter (Yan et al., 2013).

**Table 1:** **Demographics and clinical characteristics of the RRMS patients and the control subjects (two-sample *t*-test for group comparison)**.

	RRMS patients (*n* **=** 20), Mean (range)	Control subjects (*n* **=** 20), Mean (range)	*P*-values
Gender (M/F)	5/15	5/15	0.748
Age (years)	39.35 (20–57)	38.10 (22–51)	0.617
Education level (years)	11.35(6–16)	12.35(9–16)	0.322
Disease duration (months)	20.00 (3–37)	–	n/a
Normalized TWMLL (milliliter)	21.68 (0.43–51.49)	–	n/a
TF	0.88 × 10^−2^ (0.43–1.46 × 10^−2^)	1.17 × 10^−2^ (0.99–1.72 × 10^−2^)	0.10 × 10^−4^
BPF	0.83 (0.78–0.86)	0.85 (0.81–0.89)	0.49 × 10^−3^
EDSS scores	1.67 (0 – 2.5)	0	n/a
PASAT scores	84.15 (61–103)	98.60 (83–118)	0.19 × 10^−3^
MFIS-5 scores	11.15 (6–17)	0.25 (0.0–1.0)	0.10 × 10^−7^
Head motion^a^ (mm)	0.42 (0.12–0.91)	0.37 (0.13–0.84)	0.402

*BPF, brain parenchymal fraction; EDSS, expanded disability status scale; F, female; PASAT, paced auditory serial addition test; RRMS, relapsing-remitting multiple sclerosis; M, male; mm, millimeters; n/a, not applicable; MFIS-5, modified fatigue impact scale-5 items; normalized TWMLL, total white matter lesion loads in the normalized/standard brain; TF, thalamic fraction*.

*^a^Head motion according to the criteria of Van Dijk*.

#### Thalamocortical FC Analysis

The thalamic seed region was placed over the entire bilateral thalamic CDR using the Functional MRI of the Brain (FMRIB) Software Library (FSL) template (Behrens et al., 2003). (1) For FC analysis of seed-based paired ROIs, *Pearson’s* correlation (*r* value) was computed between the preprocessed average time series of seven thalamic CDRs and the average time series of the ECRs. These seven ECRs, which were previously described in detail (Behrens et al., 2003; Johansen-Berg et al., 2005), included primary motor cortex (M1), primary and secondary somatosensory cortices (S1/S2), occipital cortices, prefrontal cortex (PFC), premotor (lateral and medial) cortex (PMC), posterior parietal cortex (PPC), and temporal cortex. The ECRs were manually outlined on the MNI standard *T_*1*_*WI** using anatomical landmarks. (2) For voxel-based FC analysis, FC was calculated between the bilateral thalamic CDRs and each voxel within the ECRs. The resultant correlation coefficients were transformed into *z* scores using Fisher’s *r*-to-*z* transformation at the voxel level to better satisfy normality.

#### Statistical Analysis of FC

(1) For the FC analysis of seed-based paired ROIs, a general linear model (GLM) and the F-test was used to identify the differences in FC for thalamocortical connections between the two groups (*P* < 0.05, corrected for multiple comparisons using the Bonferroni correction) (SPSS 13.0, SPSS Inc., Chicago, IL, USA). Age, gender, BPF, and TF were considered covariates in the analyses. The alterations of seed-based FC indicate an average FC between the thalamic CDRs and the ECRs.
(2) For voxel-based FC analysis, a GLM was used in SPM8 to identify regions of altered voxel-based FC, based on one-way analysis of covariance (ANCOVA) considering age, gender, BPF, and TF as covariates followed by *post hoc* two-sample *t*-tests for between-group comparisons. For all tests, statistical significance was determined with multiple comparisons by Monte Carlo simulation (Song et al., 2011) [AlphaSim-corrected voxel-level *P*-value < 0.01 (FWHM = 6 mm, 10,000 simulations, using every ECR)] combined with different cluster sizes (calculated using AlphaSim, I ≥ 13 voxels in M1, II ≥ 17 voxels in S1/S2, III ≥ 30 voxels in the occipital region, IV ≥ 29 voxels in the PFC, V ≥ 24 voxels in the premotor region, VI ≥ 45 voxels in the PPC, and VII ≥ 30 voxels in the temporal region). The alterations of voxel-based FC indicate a regional change in the ECRs that connected with the thalamic segment.
(3) Partial correlation analysis was performed on the RRMS group to assess the relationship between clinical markers (disease duration, normalized TWMLL, BPF, EDSS, PASAT, and MFIS-5 scores) and altered FC considering age, gender, and TF as covariates of no interest (*P* < 0.05, corrected for multiple comparisons using the Bonferroni correction).

### Characterization of the Connectivity of the Thalamocortical WM Tract

#### Structural Data Preprocessing and Probabilistic Tractography

The thalamocortical WM tracts were examined to investigate thalamocortical SC, and the data processing procedure is briefly described below:

1. The diffusion data were corrected for susceptibility- and eddy current-induced distortion using the “eddy” tool of FSL v5.0^4^.
2. The “dtifit” function in the FMRIB Diffusion Toolbox (FDT v2.0^5^) was used to fit a single tensor model at each voxel of the preprocessed eddy current-corrected diffusion-weighted data.
3. Probabilistic tractography was performed using the Bayesian Estimation of Diffusion Parameters Obtained using Sampling Techniques X (BEDPOSTX) function in FDT. BEDPOSTX was used to model 5000 iterations within each voxel with a curvature threshold of 0.2, a step length of 0.5, and a maximum number of 2000 steps (Behrens et al., 2007). Pairwise ROIs, including seven thalamic CDRs and ECRs (“AND” logic), were used to calculate a distribution of fiber orientations.
4. The connectivity of long WM tracts was assigned a normalized probability value of 0.20 (Morris et al., 2008; Khalsa et al., 2013) and was visually inspected to confirm successful tracing in each individual by multiple experienced technicians.
5. Features of the long WM tracts (above threshold) connecting each paired ROIs were compared between the two groups. The considered features included the strength of a pathway, as indicated by the volume and the mean track count per WM region of the paired ROIs (Behrens et al., 2007), and above-threshold standard DTI parameters, including fractional anisotropy (FA) and three diffusivity measurements [average mean diffusivity (MD), axial diffusivity (AD), and radial diffusivity (RD)].

#### Statistical Analysis of SC

A GLM and *F*-tests with bootstrapping statistics were used to identify differences in SC of the thalamocortical tract between the two groups (*P* < 0.05, corrected for multiple comparisons) (SPSS 13.0). Age, gender, BPF, and TF were considered as covariates in the analyses. Partial correlation analysis was performed on the RRMS group to assess the relationship between clinical markers and abnormal SC considering age, gender, and TF as covariates of no interest (*P* < 0.05, corrected for multiple comparisons).

### Relationship between SC and FC

The relationship of SC–FC coupling to thalamocortical connectivity was assessed by partial correlation analysis for each pair-wise connection. Age, gender, BPF, and TF were considered covariates in these analyses.

## Results

### Demographic and Clinical Data

The demographic and clinical characteristics of the 20 right-handed RRMS patients and well-matched HCs are summarized in Table 1. Among the 20 RRMS patients, the mean EDSS score was 1.67, which corresponds to minimal disability. The RRMS patients were found to have significantly lower TF (mean ± SD: 0.88 × 10^−2^ ± 0.197 × 10^−2^) and lower BPF (mean ± SD: 0.83 ± 0.022) than the HC subjects. In RRMS patients, significantly lower PASAT scores (mean ± SD: 84.15 ± 11.92) and higher MFIS-5 scores (mean ± SD: 11.15 ± 2.96) than HC subjects indicated slowed cognitive processing and the impact of fatigue, respectively, due to MS-related damage.

### FC Measures of Thalamocortical Coherence

**Figure 1:**
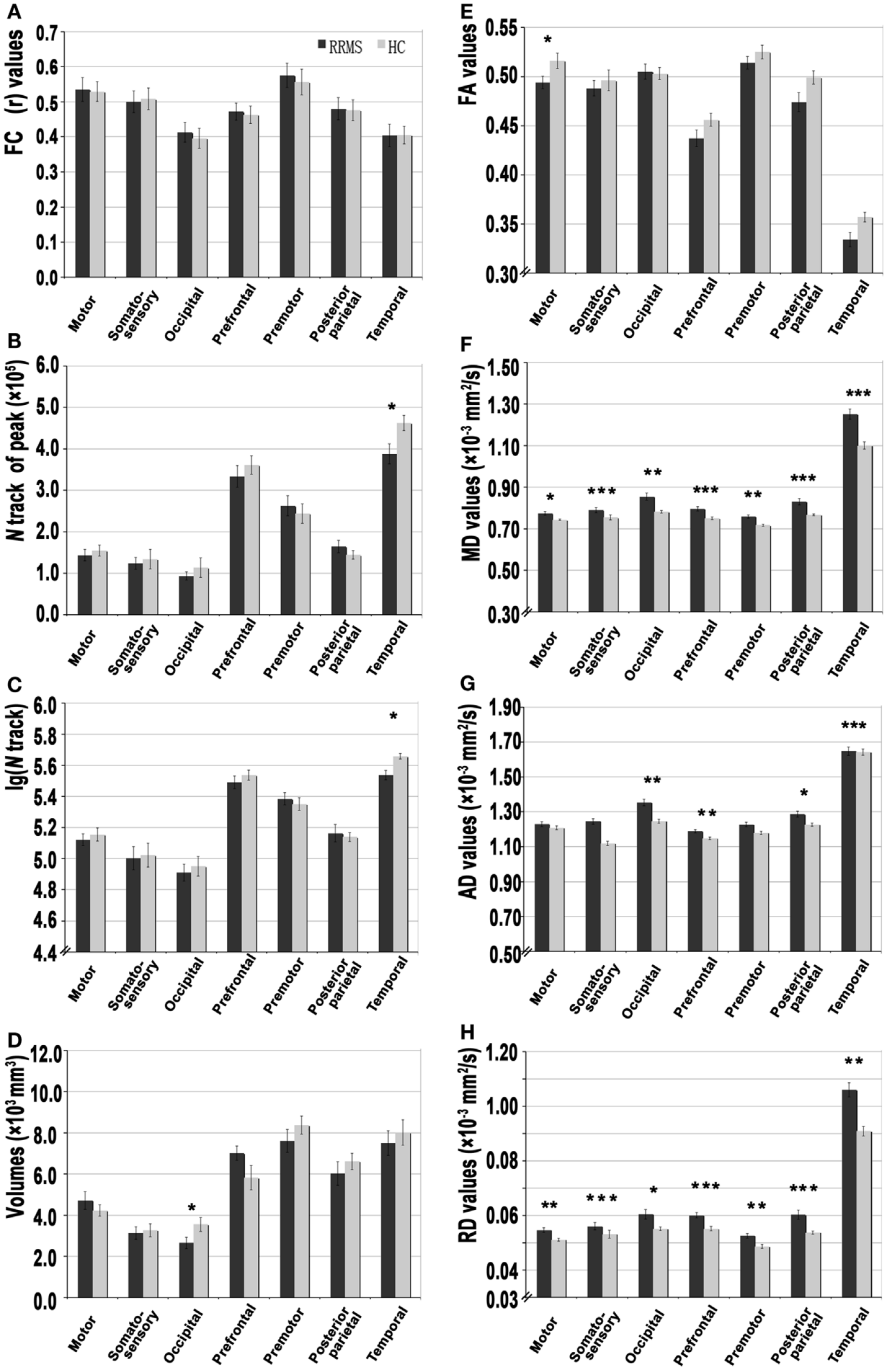
**A set of functional and structural analyses of thalamocortical connections in patients with relapsing-remitting multiple sclerosis (RRMS) and healthy control (HC) subjects**. **(A)** Resting-state functional connectivity correlation coefficient (*r* values) for each thalamocortical connection. **(B–D)** The strength of structural connectivity [based on average peak track number, volume, and log(N track)] of each connection. **(E–H)** The integrity of structural connectivity based on above-threshold (0.2) standard DTI parameters, including the fractional anisotropy (FA), mean diffusivity (MD), axial diffusivity (AD), and radial diffusivity (RD) for each thalamocortical connection (*P < 0.05, **P < 0.01, ***P < 0.001).

FC analysis of seed-based paired ROIs was used to measure the coherence between thalamic CDRs and ECRs for consistency with the SC analysis. Figure 1A presents the mean FC coefficients of the thalamic paired CDR–ECRs. In the RRMS group, there were no significant differences when compared with the HC group (corrected *P* > 0.05, Table S1 in Supplementary Material).

**Figure 2:**
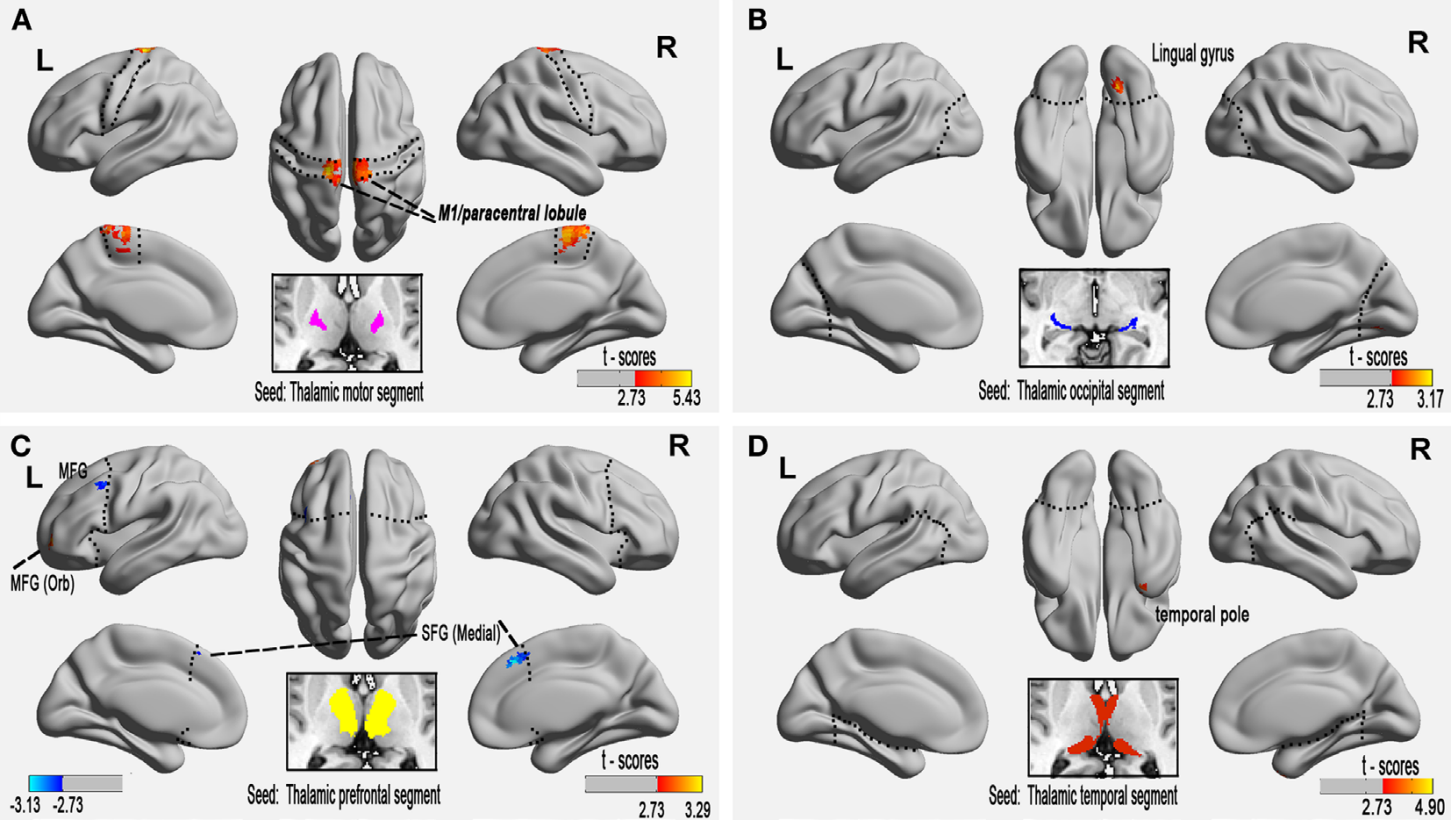
**Altered functional connectivity of the paired thalamic segment and exclusive cortical regions in relapsing-remitting multiple sclerosis (RRMS) patients at the voxel level (*P* < 0.01, AlphaSim-corrected)**. **(A)** The connectivity-defined region (CDR) and the exclusive cortical region (ECR) of the motor cortex, **(B)** occipital CDR–ECR, and **(D)** temporal CDR–ECR show only increased FC in RRMS patients. **(C)** Co-existing increased and decreased FC of the prefrontal CDR-ECR in RRMS patients.

Voxel-based FC analysis was then used to measure regional neuroplasticity in the thalamic ECRs. Compared with the HC group, the RRMS group showed significantly increased FC between the thalamus and the primary motor cortex (bilateral M1/paracentral lobule), the occipital cortex (right lingual gyrus), the PFC [left middle (orbital) frontal gyrus], and the temporal cortex (right temporal pole) (AlphaSim-corrected *P* < 0.01); the RRMS group also showed significantly decreased FC between the thalamus and the PFC [left middle frontal gyrus and bilateral superior (medial) frontal gyrus] (Figure 2; Table 2, AlphaSim-corrected *P* < 0.01).

**Table 2:** **Brain areas of altered functional connectivity in relapsing-remitting multiple sclerosis (RRMS) patients compared with healthy controls (*P* < 0.01, AlphaSim-corrected)**.

Specific cortical region	Brain regions	Peak intensity-value	Number of voxels	Peak location (MNI space)		
				*x*	*y*	*z*
**RRMS patients > healthy controls**						
Motor	Bilateral M1/paracentral lobule	5.43	321	−15	−27	75
Occipital	Right lingual gyrus	2.97	41	18	−69	−12
Prefrontal	Left middle (orbital) frontal gyrus	3.29	33	−33	60	−3
Temporal	Right temporal pole	4.90	35	33	15	−45
**RRMS patients < healthy controls**						
Prefrontal	Left middle frontal gyrus	−2.85	30	−33	18	39
	Bilateral superior (medial) frontal gyrus	−3.13	39	6	30	45

*M1, precentral gyrus; MNI, Montreal neurological institute*.

### SC Measures of Thalamocortical WM Tracts

**Figure 3:**
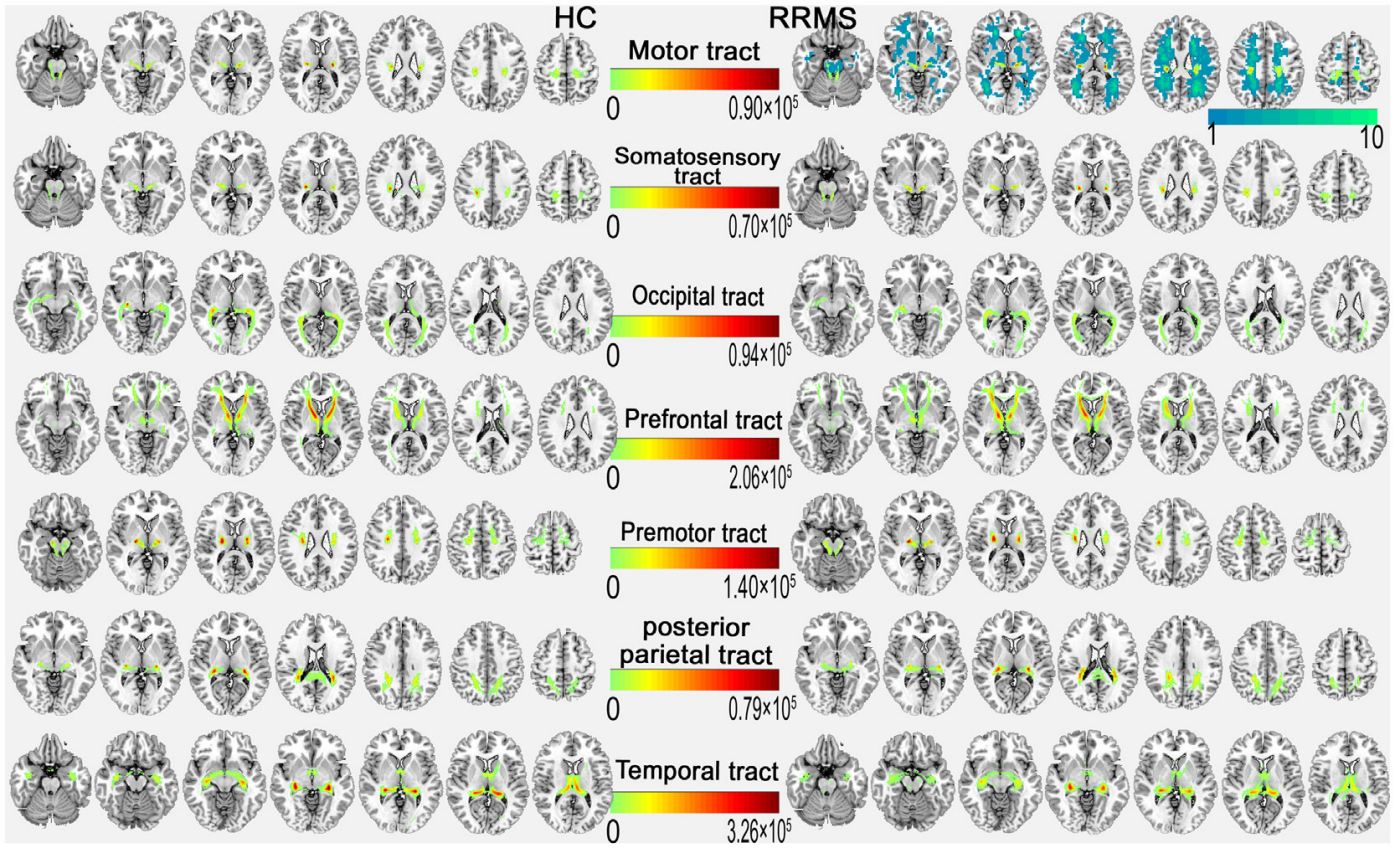
**Above-threshold reconstructed thalamocortical tracts of each coupled thalamocortical region**. In the upper right, the probabilistic tract of the motor cortex with the mean probability distribution of the white matter lesion (cold color bar on the right side) in the RRMS group is overlaid on the MNI standard T1 template in the MNI space. The color bar in the middle indicates the number of tracks (*N*).

Figure 3 shows the WM tracts identified by probabilistic tracking of thalamocortical seeds. The SC strength was assessed by measuring the mean track count, the volume, and the log(N track) along the above-threshold reconstructed tracks (Figures 1B–D; Tables S1 and S2 in Supplementary Material). In the RRMS patients, a reduced track count of the thalamocortical temporal WM tract (3.878 × 10^5^ vs. 4.623 × 10^5^) and a smaller volume of the thalamocortical occipital WM tracts (2.651 × 10^3^ mm^3^ vs. 3.555 × 10^3^ mm^3^) were detected when compared with the HC subjects.

Structural connectivity integrity were assessed by measuring the standard DTI parameters of the above-threshold reconstructed tracts (Figures 1E–H; Tables S1 and S2 in Supplementary Material). Compared with the HC subjects, in the RRMS patients, decreased FA values were detected in the thalamocortical motor, somatosensory, and prefrontal WM tracts, and increased diffusivity values for MD, AD, and RD were detected in all seven thalamocortical tracts (*P* < 0.05, Table S1 in Supplementary Material), excluding the AD values of the thalamocortical motor, somatosensory, and premotor tract.

### Partial Correlation Analysis between Clinical Markers and Abnormal Connectivity Indices in RRMS Patients

**Figure 4:**
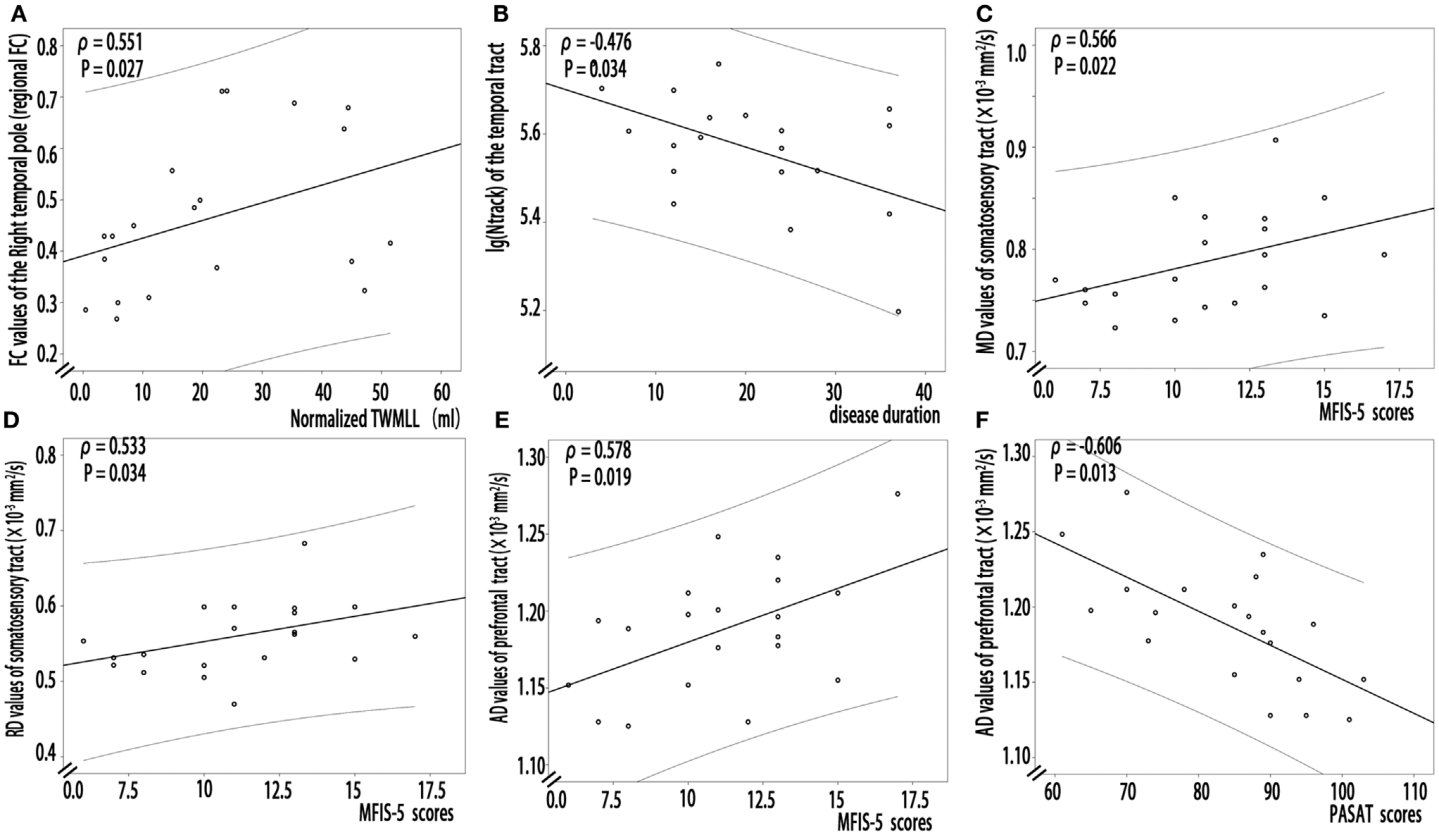
**Partial correlation analysis between structural or functional connectivity indices and clinical markers in RRMS patients**. **(A)** Increased FC of the right temporal pole positively correlated with the lesion loads (the normalized TWMLL) (ρ = 0.551, *P* = 0.027). **(B)** Decreased log(N track) of the thalamocortical temporal tract negatively correlated with disease duration (ρ = −0.476, *P* = 0.034). **(C,D)** A positive correlation was detected between the MFIS-5 score and the MD (ρ = 0.566, *P* = 0.022) and the RD values (ρ = 0.533, *P* = 0.034) of the thalamocortical somatosensory tract. **(E,F)** A relationship was detected between the AD values of the thalamocortical prefrontal tract and the PASAT (ρ = −0.606, *P* = 0.013) and MFIS-5 scores (ρ = 0.578, *P* = 0.019).

In RRMS patients, partial correlation analyses revealed that increased FC of the right temporal pole positively correlated with the lesion loads (the normalized TWMLL) (Figure 4A, ρ = 0.551, *P* = 0.027). No relationship was detected between other tracts with abnormal FC and clinical markers (*P* = 0.124−0.915).

In RRMS patients, partial correlation analyses revealed that decreased log(N track) of the thalamocortical temporal WM tract negatively correlated with disease duration (Figure 4B, ρ = −0.476, *P* = 0.034). A positive correlation was detected between the MFIS-5 score and the MD (Figure 4C, ρ = 0.566, *P* = 0.022) and the RD values for thalamocortical somatosensory WM tract (Figure 4D, ρ = 0.533, *P* = 0.034). Additionally, a relationship was detected between the AD values for the thalamocortical prefrontal WM tract and both the PASAT score (Figure 4E, ρ = −0.606, *P* = 0.013) and the MFIS-5 score (Figure 4F, ρ = 0.578, *P* = 0.019). However, in the RRMS group, partial correlation analysis revealed no significant relationship between decreased SC strength, decreased FA, or increased diffusivity values and BPF (*P*: 0.085–0.986) or the normalized TWMLL (*P* = 0.058–0.843); in addition, no significant relationship was detected between the EDSS score (*P* = 0.075–0.951) and decreased SC strength, decreased FA, or increased diffusivity values.

### Structural–Functional Coupling Measures in RRMS Patients

Partial correlation analyses did not reveal structural–functional coupling for the abnormal thalamocortical occipital connectivity in RRMS patients (*P* = 0.051–0.983).

## Discussion

In this study, we found a structural disconnectivity, as reflected by thalamocortical integrity and strength, but detected intrinsic functional plasticity, primarily reflected by regional hyperconnectivity, in minimally disabled RRMS patients. In the thalamocortical connections of RRMS patients, we found lesion load-related regional FC in the right temporal pole. We also found significant correlations of MS-related structural abnormalities with slowed cognitive processing (PASAT), the impact of fatigue (MFIS-5), and disease duration. The results of this study, which linked the demyelination-related structural disconnectivity of the long WM tract and regional functional hyperconnectivity, provide new insights into the intrinsic functional plasticity of thalamocortical connections in RRMS patients.

### Disconnectivity of Thalamocortical WM Tracts

The potential of probabilistic tractography to detect abnormalities in long WM tracts has been suggested in recent studies of patients with MS (Gorgoraptis et al., 2010; Hu et al., 2011; Zhou et al., 2014a). In the present study, connectivity-based analysis derived from probabilistic tractography between seven paired thalamic CDR and ECRs showed demyelination-related structural disconnectivity reflected by the SC integrity and strength of SC in the thalamocortical connection.

Regarding SC strength, our study revealed a decreased N track/log(N track) for the temporal thalamocortical WM tract and decreased volume of the thalamocortical occipital WM tract; this findings demonstrated decreased SC strength accompanied by a loss of long WM track quantity in patients with RRMS. Damage to the thalamocortical temporal (Bozzali et al., 2013) and occipital WM tracts (Roosendaal et al., 2009; Klistorner et al., 2015) has been identified in previous studies of MS patients. In this study, we also found disease duration-associated atrophy of the temporal thalamocortical WM tract. That is, the longer the duration of illness, the smaller the temporal thalamocortical WM tract.

In terms of SC integrity, RD reflects the restriction of water movement by both the axonal membrane and the myelin sheath of WM and is potentially useful as a marker of myelination (Klawiter et al., 2011; Janve et al., 2013). Similarly, a strong association between AD and axonal pathology was previously described in animal models (Song et al., 2003). In our study, increased diffusivity in the thalamocortical tract disruption of the integrity of axons (AD) and myelin (RD) in MS patients, this evidence supports the existence of anatomical disconnections and microstructural damage that have been reported in previous studies (Tavazzi et al., 2007; Henry et al., 2009; Gorgoraptis et al., 2010; Hawellek et al., 2011; Sbardella et al., 2013). In MS patients, WM may undergo an unpredictable combination of demyelination, gliosis, axon loss, and inflammation, which could result in FA disturbances (Bruck, 2005; Moll et al., 2011; Lassmann, 2013). This complex condition is evidenced by the different relative distributions of AD and RD, leading to apparently normal FA but increased MD values (Klistorner et al., 2015). Therefore, the FA profile may have “underestimated” microstructural alterations in the thalamocortical tract in this study. Additionally, we found that diffusivity positively correlated with the MFIS-5 scores in the somatosensory and prefrontal thalamocortical WM tracts, and these findings indicated that demyelination-related structural disconnectivity plays a major role in the impact of fatigue on physical, cognitive, and psychosocial functioning. Moreover, increased AD values for the prefrontal thalamocortical tract correlated with slowed cognitive processing (PASAT). Given that the PFC has been assumed to play a major role in the processing of complex cognitive information, the negative correlation between the AD value of prefrontal thalamocortical tract and the PASAT scores might account for the cognitive symptoms observed in RRMS patients.

### Regional Intrinsic Functional Plasticity in Thalamocortical Connections

In this study, FC analysis of seed-based paired CDR–ECRs represented a simple and straightforward FC metric of thalamocortical connections. To date, various MS-related FC alteration patterns have been reported, including decreased FC between the thalamus and several cortical regions (Liu et al., 2015b), increased FC between the thalamus and the basal ganglia and the insular, frontal, and parietal cortices, and other changes (Tona et al., 2014). Other functional MRI studies have also shown reduced distributed or regional thalamocortical FC (Liu et al., 2015b). In our study, we found this connectivity to be primarily reflected by regional hyperconnectivity in the thalamocortical connection at the voxel level (Figure 2). In MS patients, initially minimal structural damage can be sufficient to trigger a strong functional response until the exhaustion of compensation (Roosendaal et al., 2010; Faivre et al., 2012). The positive correlations observed between regional FC and the lesion load appear to demonstrate that compensatory hyperconnectivity in the right temporal pole is related to MS lesion-related demyelination (Bodini et al., 2011). Our results suggest a compensatory response maintaining function at the large-scale level due to structural damage to connecting pathways in minimally disabled patients with RRMS.

### SC–FC Coupling Measures

In general, distinct regions that are clearly and directly linked by long WM tracts also exhibit extensive functional communication (Greicius et al., 2009; Zhou et al., 2014a). This relationship pattern, SC–FC coupling, has previously been investigated using both SC and FC analyses in MS patients (Zhou et al., 2014a). In minimally disabled MS patients, a relationship between the disrupted SC and the increased FC was observed in the connections of paired DMN subregions; this result revealed a specific compensatory mechanism in response to structural damage (Zhou et al., 2014a). However, in the present study, no significant relationship between SC and FC measures was observed in the thalamocortical connection. Our finding is consistent with the results of a study by Hawellek et al. (2011), who found dissociation of the SC–FC. Although, this SC–FC relationship is inconsistent with our study of the DMN (Zhou et al., 2014a), it is plausible that this relationship is more likely to be active over time. Validating or supplementing the findings of this study is warranted in the future.

### Study Limitations

First, the examination of large ROIs from thalamic CDR–ECRs can blur the results of seed-based FC analyses. In the present study, we defined seven paired thalamic CDRs and ECRs as ROIs, which were kindly provided by Heidi Johansen-Berg and Timothy Behrens at FMRIB^6^ (Behrens et al., 2003). We also provided the results of a voxel-level analysis of thalamic FC as a supplement. Additionally, there are a few limitations to probabilistic tractography, including the possibly highly reproducible topology of the reconstructed pathways (Behrens et al., 2007; Khalsa et al., 2013) and the lack of a statistical consensus on the probabilistic tractography threshold (Morris et al., 2008). A limited assessment of cognitive function was implemented in this study. Finally, as an explorative study, our findings of altered SC and FC of thalamocortical connections warrant validation or supplementation in the future.

## Conclusion

In conclusion, compared with HC subjects, minimally disabled RRMS patients exhibited structural disconnections and regional intrinsic functional hyperconnectivity in thalamocortical system. The evidence from SC and FC analyses of the thalamocortical tract suggests a dissociated SC–FC pattern and limited regional functional plasticity to compensate for chronic demyelination-related long-distance structural disconnectivity in minimally disabled RRMS patients.

## Author Contributions

FZ, HG, and C-sZ conceived and designed the experiments; QC and BW performed the data acquisition; FZ, YP, and YZ performed the data analysis; and FZ and C-sZ wrote the paper.

## Conflict of Interest Statement

The authors declare that this research was conducted in the absence of any commercial or financial relationships that could be construed as a potential conflict of interest.

## Footnotes

^1^http://www.nitrc.org/projects/mricron

^2^http://www.fil.ion.ucl.ac.uk

^3^http://rfmri.org/DPARSF

^4^http://fsl.fmrib.ox.ac.uk/fsl/fslwiki/EDDY

^5^http://fsl.fmrib.ox.ac.uk/fsl/fslwiki/FDT

^6^http://fsl.fmrib.ox.ac.uk/fsl/fslwiki/Atlases
