# Supplementary material for: Intrinsic Functional Plasticity of the Thalamocortical System in Minimally Disabled Patients with Relapsing-Remitting Multiple Sclerosis
Source: Front Hum Neurosci. 2016 Jan 25;10:2. doi: 10.3389/fnhum.2016.00002 (PMC4725198; doi:10.3389/fnhum.2016.00002)
Supplement: Supplementary file 1 [file table_1.pdf]

Table S1 *Measures of the structural connectivity (SC) and functional connectivity (FC)of thalamocortical connection between two groups (mean ±standard deviation)*

		Motor	Somatosensory	Occipital	Prefrontal	Premotor	Posterior parietal	Temporal
Correlation coefficient	RRMS	0.535±0.155	0.499±0.138	0.413±0.125	0.472±0.109	0.575±0.153	0.480±0.140	0.404±0.146
(r- values)	HC	0.529±0.128	0.508±0.137	0.396±0.129	0.463±0.111	0.556±0.164	0.476±0.135	0.405±0.113
Mean track count(×	RRMS	1.435±0.629	1.239±0.614	0.930±0.0436	3.333±1.167	2.624±1.077	1.643±0.682	3.878±1.075
10^{36})	HC	1.547±0.594	1.338±0.572	1.134±0.106	3.607±0.932	2.441±1.058	1.438±0.458	4.623±0.833
Log(N track)	RRMS	5.122±0.173	5.003±0.337	4.911±0.246	5.491±0.181	5.384±0.177	5.163±0.254	5.537±0.135
	HC	5.155±0.185	5.023±0.344	4.951±0.285	5.537±0.145	5.351±0.179	5.139±0.128	5.658±0.081
Volumes of tract	RRMS	4.718±1.924	3.135±1.367	2.651±1.238	7.008±1.549	7.598±2.462	6.022±2.552	7.498±2.674
(×10^3 mm^3)	HC	4.229±1.240	3.272±1.435	3.555±1.542	5.819±2.652	8.370±1.975	6.614±1.802	9.013±2.767
FA values on track	RRMS	0.494±0.028	0.488±0.036	0.505±0.034	0.437±0.037	0.514±0.028	0.474±0.044	0.334±0.032
	HC	0.516±0.035	0.496±0.047	0.503±0.028	0.456±0.029	0.525±0.031	0.499±0.029	0.357±0.022
MD values on track	RRMS	0.774±0.039	0.789±0.055	0.854±0.075	0.796±0.048	0.759±0.041	0.830±0.069	1.251±0.109
	HC	0.743±0.016	0.754±0.054	0.782±0.029	0.750±0.026	0.717±0.022	0.767±0.018	1.100±0.078
AD values on track	RRMS	1.229±0.062	1.245±0.071	1.353±0.085	1.188±0.041	1.227±0.059	1.286±0.078	1.648±0.107
	HC	1.208±0.047	1.119±0.054	1.246±0.049	1.148±0.029	1.179±0.042	1.226±0.045	1.634±0.080
RD values on track	RRMS	0.546±0.036	0.560±0.058	0.604±0.078	0.600±0.060	0.525±0.041	0.602±0.078	1.059±0.115
	HC	0.511±0.027	0.531±0.068	0.550±0.031	0.551±0.034	0.486±0.029	0.537±0.023	0.908±0.079

Note: Three diffusivity measurements reported in units of×$10^{-3}$$mm^2$/s. AD = axial diffusivity, FA= fractional anisotropy, HC = healthy control, MD = mean diffusivity, RD = radial diffusivity. (Same for all tables and figures)
