# Supplementary material for: Intrinsic Functional Plasticity of the Thalamocortical System in Minimally Disabled Patients with Relapsing-Remitting Multiple Sclerosis
Source: Front Hum Neurosci. 2016 Jan 25;10:2. doi: 10.3389/fnhum.2016.00002 (PMC4725198; doi:10.3389/fnhum.2016.00002)
Supplement: Supplementary file 2 [file table_2.pdf]

***Table S2*** *General linear model (GLM) analysis and F-test for comparison of the structural connectivity (SC) and functional connectivity (FC) of thalamocortical connection between two groups [age, gender, BPF and TF were considered covariates in the analysis. F values (P values)]*

| | Motor | Somatosensory | Occipital | Prefrontal | Premotor | Posterior parietal | Temporal |
|---|---|---|---|---|---|---|---|
| Correlation coefficient | 0.473 (0.793) | 0.484(0.786) | 0.398(0.847) | 0.561(0.729) | 0.912(0.485) | 1.326(0.277) | 0.817(0.546) |
| Mean tract count | 0.480(0.788) | 0.500(0.774) | 0.288(0.917) | 1.708(0.159) | 1.194(0.333) | 0.516(0.762) | 3.366(0.014*) |
| Log(N tract) | 0.448(0.881) | 1.727(0.155) | 0.627(0.680) | 1.482(0.222) | 1.317(0.280) | 1.100(0.379) | 3.396(0.014*) |
| Volumes of track | 0.897(0.494) | 0.384(0.856) | 3.075(0.021*) | 0.647(0.665) | 1.107(0.375) | 1.280(0.295) | 0.971(0.449) |
| FA values on track | 2.503(0.049*) | 2.711(0.036) | 0.662(0.654) | 2.861(0.029) | 0.718(0.615) | 2.125(0.086) | 1.541(0.203) |
| MD values on track | 2.670(0.039*) | 6.519(0.0002***) | 4.499(0.003**) | 7.018(0.0001***) | 4.862(0.002**) | 6.809(0.0002***) | 5.996(0.0004***) |
| AD values on track | 0.509(0.767) | 2.132(0.085) | 5.208(0.001**) | 4.653(0.002**) | 2.473(0.052) | 2.640(0.040*) | 6.069(0.0004***) |
| RD values on track | 4.411(0.003**) | 6.662(0.0002***) | 3.356(0.014*) | 5.976(0.0004***) | 3.715((0.009**) | 6.317(0.0003***) | 5.509(0.001**) |

*Note: For each connection, differences at the significant level of $P < 0.05$, $P < 0.01$ and $P < 0.001$ were marked with *, ** and ***, respectively.*
